# Silk road heritage: The artistic representation of port trading culture in the images of characters in Qing Dynasty Guangzhou export paintings

**DOI:** 10.1371/journal.pone.0308309

**Published:** 2024-10-31

**Authors:** Haisa Liu, Changqing Fu, Weicong Li

**Affiliations:** 1 School of Art and Design, Guangdong University of Finance and Economics, Guangzhou, China; 2 Visual Culture Research Center, The Guangzhou Academy of Fine Arts, Guangzhou, China; 3 Faculty of Built Environment and Surveying, Universiti Teknologi Malaysia, Skudai, Johor Bahru, Malaysia; Bahir Dar University, ETHIOPIA

## Abstract

As customized artistic commodities, export paintings mirrored Guangzhou’s unique overseas trade culture and social power in a microscopic view, nevertheless, few researchers have delved into the cultural relationship between the use of the painting techniques and the characters of the export paintings. Based on an iconographic approach, this article aims to analyze the historical iconography information of the Qing dynasty export paintings from the perspective of the trade development and cultural exchange between the East and West during the 18th century by the study of the export paintings’ characters expressions and costumes colors, providing insights into the exploration of the value of tangible cultural heritage. In the process of our research, shows that: 1) the dominant laboring characters in the export paintings present standing postures, and most of them demonstrate negative emotions; 2) many of the characters of the export paintings show high similarities due to the assembly-line mode of painting during manufacture; 3) the portrayal of the characters in the export paintings reflects a return of a sense of humanity. This is evident in the fact that the subject of the paintings abounds in the scenes showing local folklore, working views, and daily life in Guangzhou. Meanwhile, the characters in the painting are vividly portrayed in a realistic style. The use of mixed colors on the costumes of different characters reflects a universal value of having regard for the equality of all men; 4) Guangzhou export painting decorative art evolution of the development of the dual development of Chinese and Western fusion of veins. Guangzhou export paintings of the Qing dynasty satisfied the customers’ reverie for ‘Oriental Civilization’ and their purpose of seeking novelty of foreign cultures. It also revealed the subtle dynamics of the interplay between Guangzhou’s social power, trading capital, and blending art.

## 1 Introduction

Visual art is an authentic reflection of the social landscape and humanities of a particular period. It has risen from a purely formal language to research of semiotic and anthropological dimensions [1]. Since the publication of UNESCO’s Text of the Convention for the Safeguarding of the Intangible Cultural Heritage in 2003 [2], attention has been paid to early-aged visual art items. In the era of lagging replicas and communication techniques, visual cultural heritages had surpassed verbal and written language in transmitting information, becoming an early source of people’s acknowledging the past and experiencing traditions. The authors of this article follow closely the continuation of Western-style art in the creation of characters for Guangzhou export paintings, which serve as visual evidence of the merging of Chinese and Western art and culture of the Silk Road.

Early export paintings were called ‘foreign paintings’ by the painters themselves and ‘Chinese paintings’ by the foreign buyers. By the mid-20th century, Western art history referred to them as ‘Chinese export paintings’ or ‘Chinese trade paintings’. By now, the term ‘export painting’ has been clearly defined and universally accepted: paintings created in China between the 18th and early 20th centuries in places such as Guangzhou and sold mainly to foreign merchants and tourists [3]. Chinese paintings that were collected abroad, not purchased as commodities, do not fall into the category of export paintings [4]. There are as many as nine types of export paintings, covering a wide range of subjects, including politics, economics, military, religion, social life, folklore, and natural creatures of the time [5].

There is a need for an explanation of how the Maritime Silk Road promoted the Eastward Expansion of Western Painting Style and the uniqueness of the Ming and Qing dynasties in this study. As a very special art commodity, export paintings became tangible carriers to extend the Baroque and Rococo styles in Chinese painting in the 18th century [6]. With the opening of the New Passage in the 15th century, Portugal gradually dominated business trading [7] between Europe and the Far East, thus helping to sell products from East to West, while at the same time, transferring technology from West to East. By the late 17th century, as cultural media, Chinese export paintings were already exported to Spain, the Netherlands, and Great Britain [8], bringing the oriental cultural concept to the European art field. A hub of the Maritime Silk Road Guangzhou was, export paintings during the Ming and Qing dynasties reached its prime in this area by combining both Chinese and Western art forms [9].

With the rise of the cultural heritage economy in modern times [10], export paintings, with only social function left, have gradually been marginalized. Lacking material to study artistic characteristics, shallow digging into its historical value, weak preservation of this heritage, and shortage of innovation in visual elements and applications are aspects that reflect the insufficiency in studying Guangzhou export trading culture. In the Chinese variety show National Treasure aired in 2018, Bai, the director of the Display and Exhibition Center in Guangdong Museum pointed up the cultural value of Guangzhou export paintings and the importance of being visual evidence to experience historical humanistic landscapes [11]. In China’s 14th Five-Year Plan for Cultural Development published in 2021, the issue of preservation, inheritance, and restoration of marine culture has been emphasized to further adapt to meet the needs of the new era. It also reaffirmed that cultural heritage is the footstone of the spirit of contemporary artworks and innovations [12]. The protection, inheritance, and restoration of marine cultural heritage have been enhanced in the latest ‘14th Five-Year’ cultural development plan, yet efforts in the preservation of exported cultural relics and cultural heritage remain insufficient. Contemporary cultural restoration has ascended to the level of protecting outstanding cultural phenomena [13], with a greater emphasis on uncovering and disseminating excellent cultural genes [14], to meet the demands of the new era and invigorate new vitality [15].

As historical evidence of East-West trading as well as the blending of Eastern and Western cultures in the 18th century Maritime Silk Road, export paintings fulfilled Westerners’ imagination and appreciation of "Oriental civilization". With these images, Westerners could follow the lead to get a glimpse of the mysterious ancient oriental country and its glamour. Scholars in China and abroad have discussed the export paintings focusing on their "economic attributes" and "social attributes" respectively.

On the economic aspect, Kleutghen [16] explored the economic attributes of the production and distribution of hand-painted wallpaper, a distinct category of export paintings, arguing that these Chinese wallpaper themes, which were popular in Europe, reflect class differences with a Western aesthetic narrating style. Bruijn [17] found out that as export hand-painted wallpaper painting techniques improved and the content became closer to real life, the early exclusive-customized and supplied hand-painted wallpaper was no longer a privilege to the British and Irish aristocrats. During the same period, the decreasing production cost of hand-painted wallpaper further led to innovation in designs such as patterns, that adapted themselves to the interior decoration at that time, which gained great popularity in the British, Dutch, and American traders coming to China from the 1840s to 1890s. Bruijn [18] found that the commercial profits had a significant impact on its design and argued that Chinese export hand-painted wallpaper exported to Europe retained its oriental style, while as a commodity, it originated commercially rather than culturally. Sevänen [19] held the opinion that the European art concept of the 18th century was developing a tendency to be more independent and rational. Comparably, Chinese traditional painting at that time held very little influence on the export of hand-painted wallpaper. Chinese traditional painting maintained a high social status and development during that period.

On the level of social attributes, Wu [20] found that to some extent, Chinese export wallpaper mirrored the colonial economic control and political supremacy. By the political complexion, westerners used these paintings to image their experiences and visualization of oriental culture. Rostislav [21] pointed out that the Qing Dynasty export wallpaper’s target customers were European aristocrats and the elite class. Subjects of realistic and vivid city scenes were commonly seen in export wallpaper. Jiang’s [22] research found that, compared with painting skills, wallpaper trading in China and Britain before the Opium War emphasis more on the gradation in color and changes of patterns, which was possibly caused by the differences in the market’s demand and aesthetic values of these two countries. Bevan et al. [23] also marked that the Chinese style wallpaper in British during the 18th century had fulfilled the European’s fantasy of the Oriental culture, while also indicating the social class and status differences in British. McClintock et al. [24] claimed that the popularity of China’s export wallpaper was not only driven by commercial needs but also motivated by a desire and demand for foreign culture experience, therefore, it reflected the changes and alternations of the social culture.

Research in this field has shifted focuses from artistic form to its content, and to the cultural connotation as the study deepened, and further brought up to the level of national identity and cross-cultural communication. Two parallel perspectives emerged in discussing the export paintings: 1) commercial attributes. It’s a mixture weaved by capital and art in the context of the cultural collision of Sino-foreign trading. It revealed how a commercial art market of cultural fusion between China and the West had formed in Qing Dynasty Guangzhou. 2) social attributes. It is a material cultural phenomena developed along with the society, which serves as an epitome of several social phenomenon such as social status, social class polarization, aesthetic taste, and life of the time. By now, few scholars have put character images under the microscope, to discuss this visual symbolic language under the context of its multi-dimensional attributions in history, society, culture, and economics, as well as its paradigm developments over time. This is where the value of this study lies, as we’re going to dig into these issues.

The study of the painting art of characters in Guangzhou export paintings of the Qing Dynasty helps to sort out the characteristics in creating the Guangzhou export paintings during the Ming and Qing Dynasties, excavate the cultural connotations and artistic values behind the cross-cultural combination and its evolution of historical iconography, and analyze the emerging cultural relationships behind the decorative art. In the process of mutual appreciation between Eastern and Western civilizations, export paintings carry an important role in drawing history with images, proving history with images, and proving images with history. Relevant series of books, museum collections, and archive resources have integrated most of the information on Qing dynasty export paintings, which is an important basic study material for this paper.

## 2 Methodology

### 2.1 Influence of the Qing Dynasty period on export paintings

The Qing Dynasty (1644–1911) marked a significant era in Chinese history, characterized by pivotal political, economic, and cultural developments. From the mid-17th century, China implemented a series of maritime prohibition policies aimed at regulating foreign trade and protecting the domestic economy from external influences [25]. However, due to internal and external pressures and economic necessities, the Qing government gradually relaxed these restrictions [26]. With the establishment of the Cantonese Thirteen Hongs in 1757, which served as the government-designated monopoly for foreign trade, Guangzhou emerged as the sole trading port for China’s foreign trade [27]. As a hub for commercial trade and multicultural integration, the unique historical context of Guangzhou in the 18th century provided a crucial social and material foundation for the production and evolution of export paintings, imbuing them with significant economic implications [28].

### 2.2 Research methodology and procedure

This paper combines iconography and literature analysis approaches. By sorting and analyzing sample data, we first gain insight into the potential correlation between statistical data and Guangzhou trade culture. Then, based on the literature review and data re-organization, we chase down the influencing factors that affect the decorative characteristics of export paintings in the Qing Dynasty to construct the correlation between the process of the merging of Chinese and Western cultures and the development of the art forms of export painting. Thirdly, we make an assay of the artistic style of export paintings focusing on both character images and their costume colors, to provide explanatory historical analysis by offering historical references. Lastly, we would take out an in-depth interpretation of the cultural shaping relationship behind the development of character images in export paintings, and reveal the reflected cultural phenomenon.

#### 2.2.1 Sources and statistics

**Fig 1:**
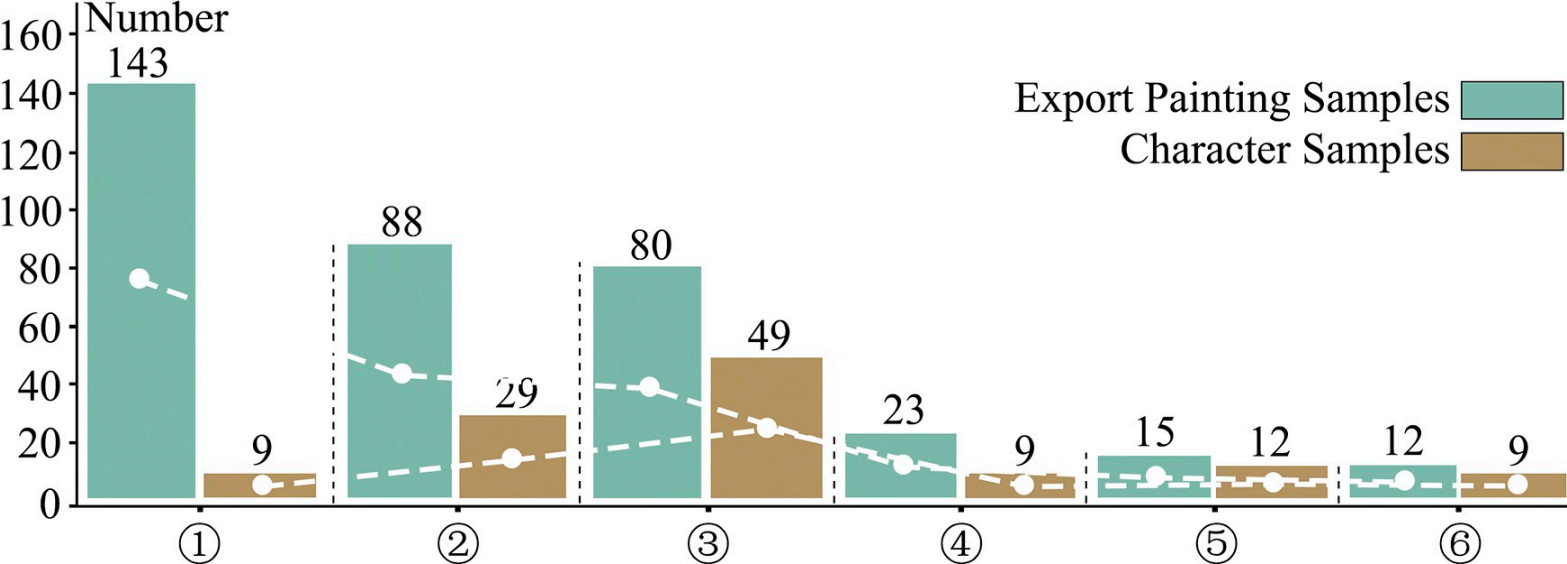
Statistical comparison of number of export paintings and characters (Source: Own statistics).

The empirical evidence of this article comes from 361 export paintings in Chinese Natural History Paintings (17 volumes), most of which are currently collected by the National Library of France and The University of Manchester Library [29, 30]. The above samples are all gouache paintings signed and dated from the 18th to 19th century during the time of the ban on maritime trade. **Table 1** displays basic information on these samples. Classified as a particular category according to the artistic form, these samples show a rich variety in terms of subjects as well as contents. Among the samples collected **(Fig 1)**, 143 (39.6%) of the artworks belong to the "Plants, Birds, Flowers, and General" album, 88 (24.3%) are from "Porcelain Manufacturing and Trading," and 80 (22.1%) originate from the "Tea Picking, Planting, Production, and Trade" album. The samples presented indicate the number of samples collected by our team, while not demonstrating the actual number the volumes include. A list of sample data can be found in the **S1 Appendix**.

**Table 1:** Data sources of export paintings and characters.

Code	Volumes	Number	Description
①	Plants, Birds and Flowers and General	1	Predominantly flower and bird paintings, containing a few figures, insects, roof designs and botanical white drawings.
②	Porcelain manufacturing and trading	2	It reveals the fine process of porcelain production and the prosperous scene of export trade at the end of the Qing Dynasty, while recording the folk activities associated with it.
③	Tea picking, planting, production and trade	1	It shows the whole process of tea picking, tea planting, tea making to trade in China in the 18th century.
④	Peasant farming	1	Depicts the daily life and farming practices of China’s agricultural society.
⑤	Varnish Atlas	2	Demonstrates the fine process and varied applications of Chinese varnish craftsmanship in the 18th century.
⑥	Cotton production	1	Documents the various stages of the cotton weaving process in 18th-century China, for example, from the cultivation of cotton to its weaving into cloth.
⑦	Silk production	1	Shows the detailed process of silk production, e.g., from silkworm rearing to silk extraction.
⑧	Tea View Panorama	1	A comprehensive description of the many facets of tea making and tea art life.
⑨	Metal Crafts White Drawings	1	Depicts the fine art of metalworking and the production of decorative objects in 19th century China.
⑩	Tea and Pottery, People, Boats, Plants and Birds	1	A comprehensive display of traditional crafts such as tea making and pottery making, as well as the lives of the people associated with them, transportation and natural landscapes.
⑪	Zoological atlas	1	Characterizes some of the animals native to China in the late 18th century.
⑫	Atlas of Chinese Plants	1	Records of plant species endemic to China in the 19th century and their applications.
⑬	Atlas of Chinese Medicinal Herbs	1	Types and pictures of herbs are depicted through drawings.
⑭	Cotton, glass, paper making, coal mining	1	Documents the production process and application of industrial materials in 19th century China.
⑮	Gouache on pith paper	1	Comprehensive scenes covering 18th century Chinese city life.

Source: own statistics

In the 361 collected export paintings, the same image could commonly appear in different scenes and serve as different characters (e.g., farmer, official, painter, tea merchant, monk, etc.). In this paper, 117 characters are extracted from the painting in an integrative consideration of the frequency of their appearances, the distinctiveness of their occupations, the artistry of the painting, and the style of drawing. **Fig 1** shows the number of character samples that are collected from the six volumes. Since Volume① contains only a few characters, while Volume④, ⑤, and ⑥ mostly depict sceneries, the major characters we choose to be samples are from Volume ② and ③ (for the richness and diversity of characters).

**Fig 2:**
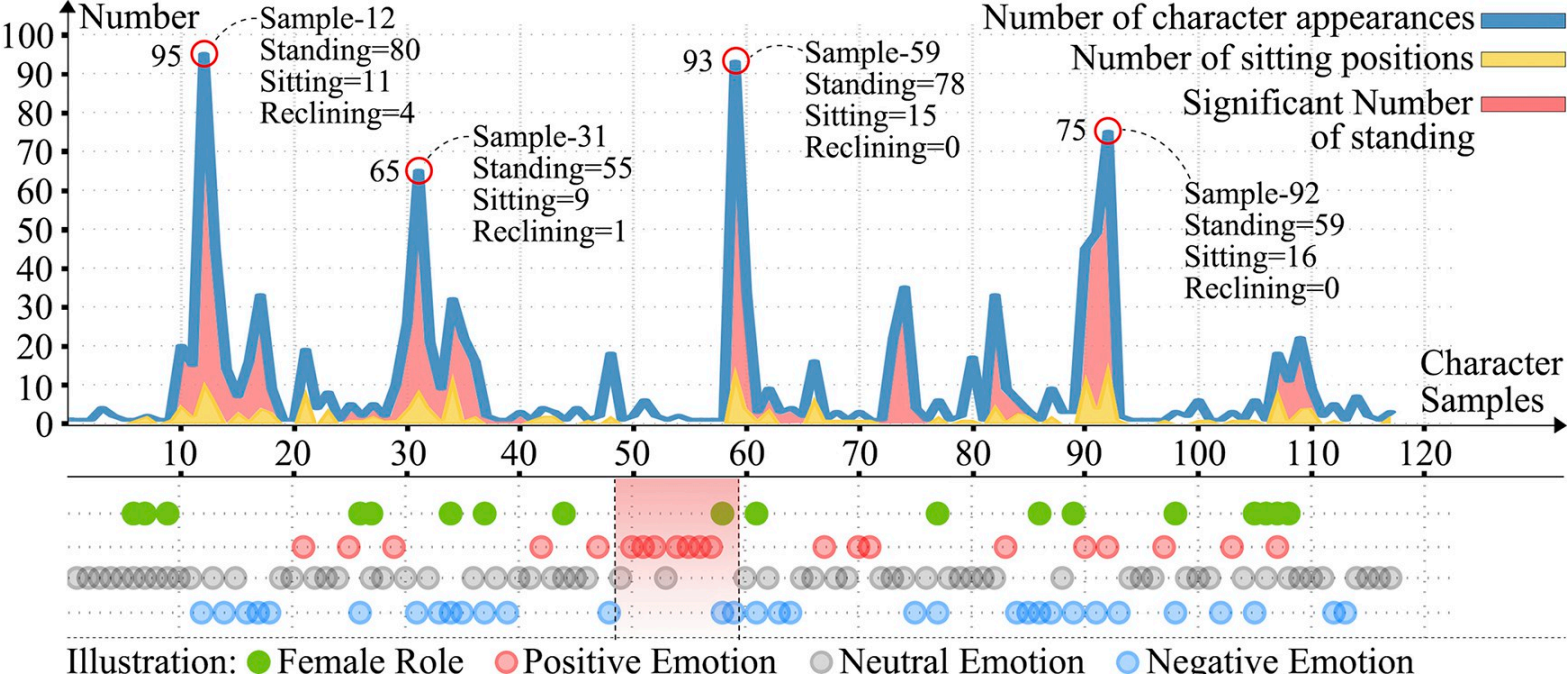
Statistics of 117 character samples in 361 export paintings (Source: Own statistics).

**Fig 2** compares the frequency of appearance of every character sample (blue line) and female character (green dots), as well as the number of characters in different positions (marked with red and yellow lines, respectively). Characters samples 12, 31, 59, and 92 appeared frequently, and their emotional appearances were marked by red, grey, and blue circles, corresponding to positive, neutral, and negative tendencies, respectively. Characters showing positive emotions are concentrated in the red region (samples 47–57). **Table 2** records the basic information of the 15 representative characters in **Fig 2**, revealing the fact of the dominance of male characters in Guangzhou’s trading society. The characters of the largest number are laboring people (attendants, boatmen, farmers, porters, and tea workers), and these characters are usually portrayed in obedient, loyal, and passive manner. In the tea-processing and shipping scenes, tea merchants and shipowners are depicted with stylish, elegant grace and energetic spirit, which to some extent reflects the social class features. Samples 51 and 54–57 also show positive spirits, yet their expressions reflect more of the nature of their occupation as entertainers than the social reality. Sample 92, a porter character, shows exceptional cheerfulness as a member of the bottom of society, which may imply a social appreciation for hard work and a profound influence cast upon social structure and value by trade and business culture. For example, most of the traditional Chinese paintings are based on landscape and natural elements, while some of them are dominated by aristocratic and upper-class themes, with fewer marketplace subjects. The development of trade and culture has led to a gradual diversification of the themes of export paintings and has shown the labor spirit and value of the lower classes to the world, reflecting the recognition of the dignity of labor.

**Table 2:** Basic information of 15 representative characters.

Sample	Occupation	Number	Emotion	Gender	Scenery	Demeanor	Posture	Source
12	Attendant	95	↓	Male	Touring the Tea Plantation	Exhausted	Standing	③
31	Farmer	65	↓	Male	Timber Felling	Discouraged	Standing	③
47	Foreign Tea Merchant	2	↑	Male	Supervising Work	Elegant	Standing	③
48	Tea Worker	18	↓	Male	Processing Tea	Distressed	Standing	③
49	Sedan Chairman	1	○	Male	Visiting officials	Respectful	Standing	③
50	Shipowner	2	↑	Male	Receiving and inspecting goods	Agreeable	Reclining	③
51	Performer	6	↑	Male	Theatre performance	Happy	Standing	③
52	Performer	2	○	Male	Theatre performance	Contemplative	Sitting	③
53	Audience	1	○	Male	Theatre performance	Calm	Raising Head	③
54	Performer	2	↑	Male	Theatre performance	Delighted	Standing	③
55	Performer	1	↑	Male	Celebrating Festival	Joyful	Standing	③
56	Performer	1	↑	Male	Celebrating Festival	Proud	Standing	③
57	Performer	1	↑	Male	Celebrating Festival	Confident	Standing	③
59	Boatman	93	↓	Male	Delivering mud and stones	Nervous	Sitting	②
92	Porter	75	↑	Male	Delivering China clay	Leisurely	Standing	②

Illustration: ↑Positive ↓Negative ○Neutral

Source: own statistics

### 2.2 Correlates factors affecting the decorative art of Guangzhou export paintings in the Qing Dynasty

Drawing upon a literature review, this study systematically examines the art characteristics of export paintings through eight dimensions: composition, subject, color, material, technique, pattern, craft, and scale. It elaborates on how these artistic features are influenced by economic (▲), political (▼), religious (●), traditional Chinese thought (◆), Western culture (■), function (○), and aesthetics (□). **Table 3** presents the specific characteristics of export paintings from Qing Dynasty Guangzhou and the associated influencing factors, with different symbols representing various societal factors.

**Table 3:** List of influencing factors on the decorative art of Guangzhou export paintings in the Qing Dynasty.

Artistic Element	Findings	Relevance
Composition	Unlike traditional Chinese paintings, the "focal point perspective" is adopted, while the use of light and dark compositional relationships creates a three-dimensional space in order to objectively reproduce the three-dimensionality and realism of the scene in the physical space.	◆■□
Subject	In order to satisfy the curiosity of Western scholars, Guangzhou export paintings were based on images of scenes from different seasons and real-life situations.	◆■
Color	The color construction of Guangzhou export paintings shows a westernized trend (realism), which is reflected in the color activity and strong contrast between light and dark.	◆■
Material	Chinese painters in painting the expression of exotic decorative flowers and birds wallpaper, both the use of stone green, cinnabar, lead white, vine yellow, magenta and other traditional Chinese paintings of natural mineral pigments, but also the use of ivory white, Prussian blue, peacock blue, rose red, royal yellow, such as bright fancy imported gouache, watercolor pigments.	▲
Technique	Traditional Chinese brush painting techniques such as outlining, draining and coloring are used, as well as traditional Chinese painting methods of spatial expression such as "white space", "superimposition" and "reality and emptiness" to embody the subtle, aesthetic and elegant oriental meaning.	■
Pattern	Painters will use woodcutting triangular knives or woodcutting diagonal knives on pear wood or jujube wood to carve the outline lines of figures, birds and flowers, landscapes and other images according to the pastel, and then brush the ink onto the carved molds, ink the topography to the special paper to print out the figures or birds and flowers and the outlines of the gardens and buildings, and the whole set of procedures is similar to the production of the traditional woodblock New Year’s Paintings.	▼◆■□
Craft	Combined with the western painting, the color halo reflects the relationship between light and shadow, as well as the shaping of space.	▲
Scale	Guangzhou Export Painting adopts the western perspective of observation when outlining the scale of the picture. When observing a specific object, one can not only see the shape, color, and size of the object, but also feel the contrast between light and darkness shed on the object by natural light, thus feeling the orientation of the object in space and the spatial distance between the observer and the object.	◆■○

**Source**: own statistics

References: [3, 10, 31–39]

In terms of artistic creation, the export paintings chose subjects showing ordinary people living their normal lives, and the using of caustic perspective and three-dimensional expressive techniques indicates influences of the trading business development and socio-economic factors at that time. At the same time, it also reflects the combination and collision of Chinese and Western cultures. The elements mentioned above are closely related, which produces good value for research.

## 3 Results

### 3.1 Social metaphors: An economic approach of thinking hinted by portrait duplication phenomenon

To pore deeply the origin of Guangzhou export paintings in the Qing Dynasty, it’s crucial to find out the social and cultural factors behind the artistic representations rather than merely following clues to interpret how this art performs and the artistic style.

**Fig 3:**
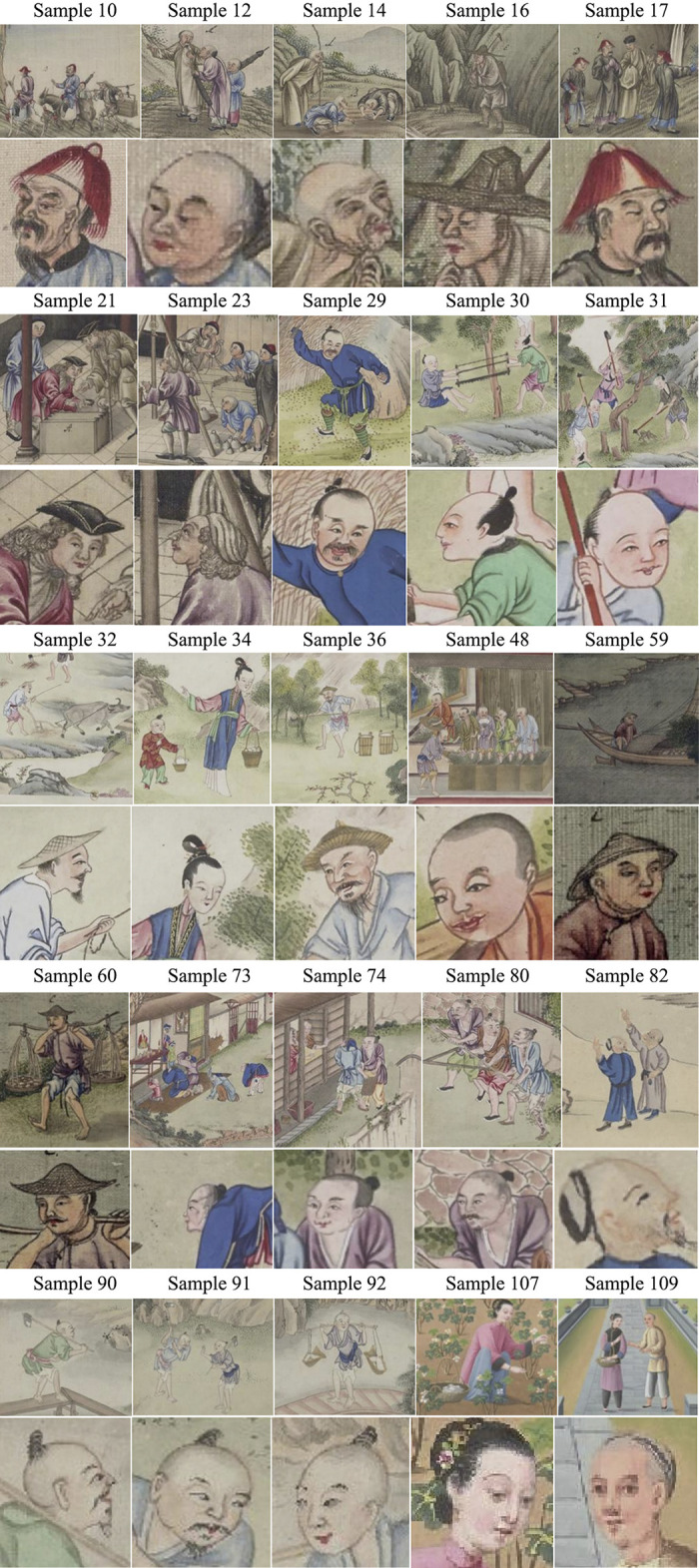
25 characters of samples in Table 4.

Given the limitations of capacity of this paper, our team has analyzed 25 characters with a frequency of appearance greater than 10 times among 117 characters. Characters in **Table 4 and Fig 3** offer a great variety of occupations and richness in gestures, which in a way, reflect truthfully the complexity and plurality of Guangzhou’s social and commercial structures.

**Table 4:** 25 characters with appearance greater than 10 times (Source: Own statistics).

Sample	10	12	14	16	17
Character	Official	Attendant	Monk	Monk	Official
Scene	Tea Delivering	Tea Garden Overseeing	Tea Inspecting	Tea Collecting	Inspecting
Demeanor	Sturdy	Tired	Calm	Haggard	Serious
Emotion	○	↓	○	↓	○
Frequency	20	95	14	16	33
Source	③	③	③	③	③
Sample	21	23	29	30	31
Career	Foreign Merchant	Foreign Merchant	Farmer	Logger	Logger
Scene	Picking Tea	Weighting Tea	Driving Out Beasts	Clearing A Wild Area	Clearing A Wild Area
Demeanor	Calm	Calm	Panic	Calm	Steady
Emotion	○	○	↓	○	○
Frequency	19	10	10	26	65
Source	③	③	③	③	③
Sample	32	34	36	48	59
Career	Farmer	Tea Picker	Tea Picker	Tea Processer	Boatman
Scene	Farming	Delivering Tea	Watering Tea Plant	Processing Tea	Delivering Porcelain
Demeanor	Cheerful	Focused	Calm	Haggard	Distressed
Emotion	↑	○	○	↓	↓
Frequency	21	32	16	18	93
Source	③	③	③	③	②
Sample	60	73	74	80	82
Career	Porter	Civilian	Civilian	Farmer	Farmer
Scene	Carrying Mud and Stones	Praying For Blessings	Paying Grain Tax	Raising Bran	Inspecting Lacquer Trees
Demeanor	Tired	Devotional	Distressed	Calm	Joyful
Emotion	↓	○	↓	○	↑
Frequency	34	22	35	17	33
Source	②	④	④	④	⑤
Sample	90	91	92	107	109
Career	Quarryman	Quarryman	Porter	Cotton Picker	Cotton Picker
Scene	Quarrying	Quarrying	Delivering	Picking Cotton	Chatting
	Porcelain	Porcelain	Porcelain		
	Stones	Stones			
Demeanor	Happy	Confused	Optimistic	Focused	Pleasing
Emotion	↑	↓	↑	○	↑
Frequency	45	49	75	18	22
Source	②	②	②	⑥	⑥

Illustration: ↑Positive ↓Negative ○Neutral

Data: from own statistics

Figures: from the book of Chinese Natural History Paintings

Link: https://www.aliyundrive.com/s/xAoLuHTP4Li/folder/62c7f16b82011dc014ff4d3bb3c5e82fb4c309af

Source: Figshare Website

URL: https://doi.org/10.6084/m9.figshare.25511146.v1

Search date: 25 February, 2024

Copyright: Creative Commons Attribution 4.0 International (CC BY 4.0)

**Fig 4:**
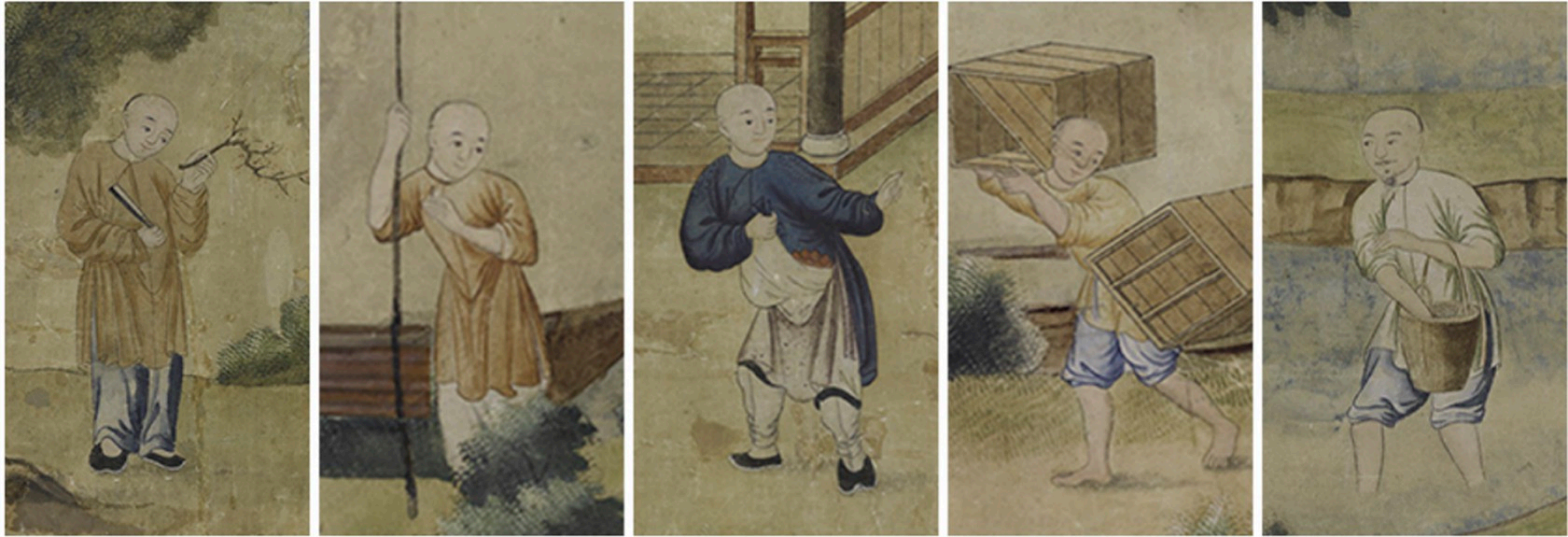
Scenes of painting in Guangzhou export painting studios and character images in the artworks. **Figure name**: -Qing Dynasty Qianlong Period Agricultural and Commercial Scenes Export Wallpaper (清乾隆农耕商贸图). Author: a-anonymous; b-anonymous. Source: http://www.gdmuseum.com. Copyright: Guangdong Museum, China. Use situation: use the original picture without any modification. Terms of use: Any unit or individual who uses the contents of this website by reprinting, quoting, extracting or downloading shall indicate the author, and the source of the pictures and articles shall be "Guangdong Museum Website" or http://www.gdmuseum.com. Without the written permission of the Guangdong Provincial Museum or the relevant obligee, the content used shall not be modified.

Description: We found some ancient Chinese and European fine art paintings shared by Kailin Huang under CC BY 4.0 from the Figshare data sharing platform. The author acknowledges that these works are in the public domain and are in accordance with the ’Berne Convention for the Protection of Literary and Artistic Works. ’Chinese Natural History Paintings’, a series of books that are used in Table 4 and Fig 4 of our manuscript.

Citations: Huang, Kailin (2016). Window on Culture: Appreciation of Chinese and Western Painting Art. figshare. Dataset. https://doi.org/10.6084/m9.figshare.25511146.v1

The observed similarity and uniformity of the character images in export paintings is an indication mark of the commercialization of Qing Dynasty Guangzhou export paintings. It is a combined result that was caused by standardized production in the individual painting workshop in Thirteen Hongs, and the concentration of social cultural assets. According to **Table 4**, characters of six specific occupations (attendant, logger, boatman, tea picker, porter, and quarryman) show up more than 30 times. Its re-appearances declare a preference for certain kinds of social roles. Among these, sample 12 (attendant) and sample 59 make over 90 times of presence, followed by sample 31 (65 times), sample 90 (45 times), and sample 91 (49 times). This high repetition of certain occupations does not fit in the common logic of traditional Chinese painting creations.

Based on incomplete statistics, the number of Guangzhou export paintings in the late Qing Dynasty reached as high as 16,969 pieces [40, 41]. The soaring number of orders posed a challenge to the painters, who soon faced a highly increased cost of manufacture and workload. To finish the burdening customized commissions, painting workshops in Guangzhou attempted a mechanical way to produce export paintings by employing a lot of painters [42] (**Fig 4**). A picture was divided into different units and different parts and different painters collaborated to finish drawing beautiful and vivid scenes within a very limited time. Each painter had an outline sketchbook as a reference, and they would select sample references from this book and manage to work together on copying and detailing the wallpaper according to the employer’s requirements, forming an efficient production process. Over time, the techniques and styles of different painters tied together with high similarities, thus creating a picture of a unified style. This is just the reason why the same character was repeatedly portrayed in different subjects of export paintings. The way of producing had led to the likeness and routinization of the wallpaper. As you may see, the characters in Farming and Trade Export Wallpaper in the Qianlong Period of the Qing Dynasty demonstrate the same gestures, facial expressions, and costumes [42] (**Fig 4**).

The delicate depiction of the characters’ body movements and facial expressions, as well as their occupations and appearance frequency, reflect to a certain extent the return of humanity in the creative concept. The drawing of characters’ facial expressions is by the standard of traditional Chinese court painting of showing elegant, reserved, and noble fashion of the appearance of the figure, while also demonstrating humanistic compassion. 12 (48%) characters are depicted in a moderate manner of the samples counted. 7 (28%) laboring people (attendants, monks, tea processors, porters, boatmen, civilians, and quarrymen) express declining spirit such as tiresome, haggardness, and distress, indicating their suffering of hardness and pressure as lower ranks in the society. For instance, a boatman (sample 59) on an isolated boat on the river echoes with his confused and glooming look, demonstrating the helplessness towards the hard life of the laboring people; a quarryman (sample 90) is presented in an excited mood after discovering rare mineral, symbolizing their wistfulness for a turn for the better life. Nevertheless, 6 (24%) characters of the underclass (farmer, civilian, quarryman, porter, cotton picker) are brought out cheerfully, revealing their persistence and dauntlessness towards the hard life. For example, a praying civilian (sample 73) shows up 22 times in multiple export paintings, showing the wishes for a good life of the lower ranks. Furthermore, the scarcity of female characters gives prominence to their marginalization in the social structure and imbalance in the social relationships. There’s also a noteworthy fact that samples 107 and 109 are distinctly different from the other samples, with recognizable light and shade contrast of portraying characters’ faces, and three-dimensional perspectives in shaping, which embodies the merging of Chinese and Western art forms.

### 3.2 Cultural connotation: The fusion of Chinese and Western art of the application of Chinese primary colors and secondary colors

The change of use from Chinese primary colors to secondary colors in export paintings implies the social innovation from complying with the traditional code of ethics to more open-minded inclinations, which are also influenced by the combination of Chinese and Western arts and cultures. The use of colors of the characters’ costumes in export paintings is the embodiment of this tendency.

Chinese traditional primary colors refer to five pure colors of indigo, red, yellow, white, and black (may also include other unmixed colors), and on the contrary, secondary colors refer to colors mixed with primary colors [43]. In ancient China, the use of color of costumes followed a strict set of etiquettes, and the colors of the uniforms could be used to make distinctions on the officials’ power and rank [44]. Therefore, the traditional primary colors and secondary colors became the tools for identifying the noble and the underclass, classifying social ranks, thus the usages should not be mixed in any way [45]. In Chinese traditional paintings, primary colors are often used on painting characters, while secondary colors are seldom for their demeaning undertone. It mirrored the social value of self-restraint and following social norms as well as class differentiation.

**Fig 5:**
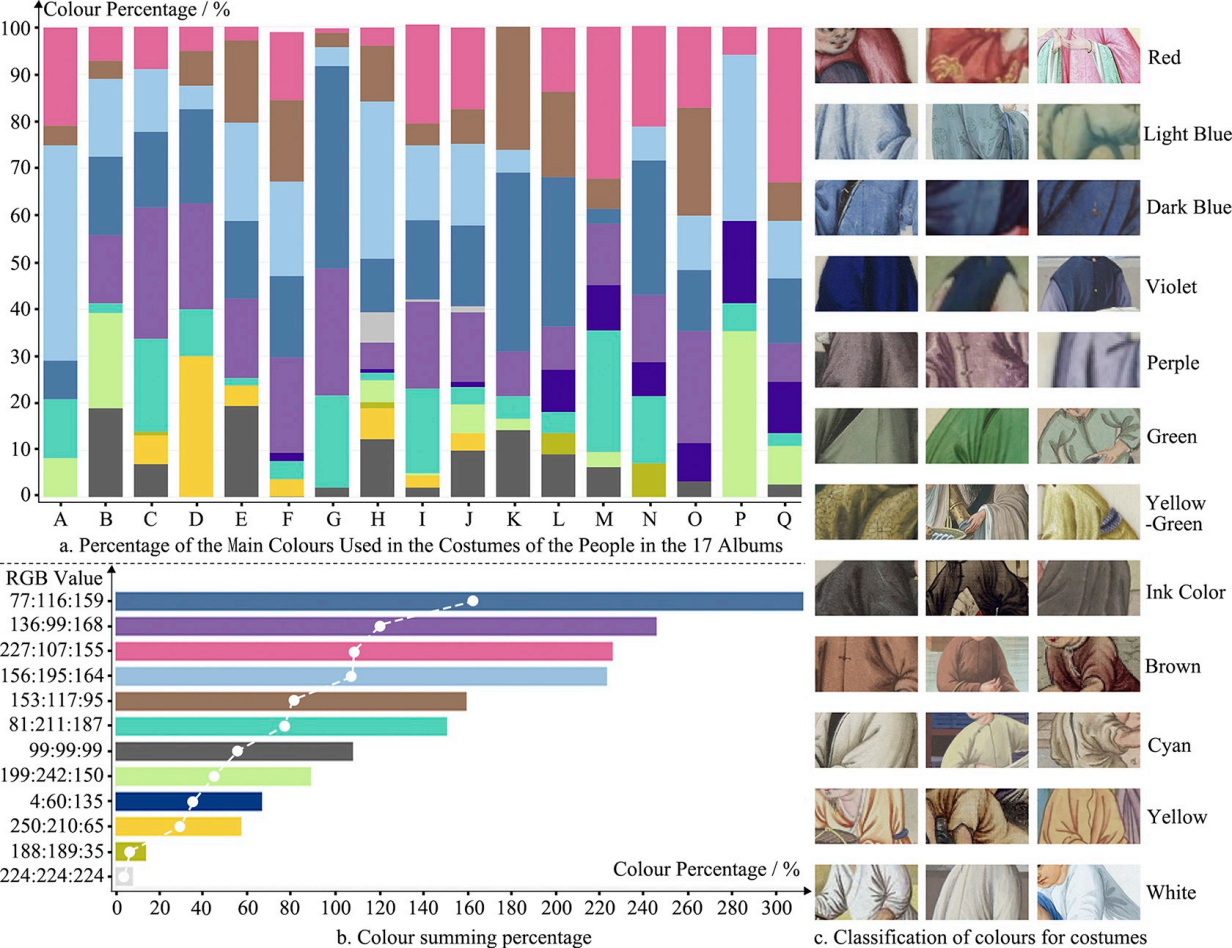
Color analysis of characters’s costumes in the 17 albums of chinese natural history paintings (Source: Own statistics). Date: from own statistics. Fig 4: from the book of Chinese Natural History Paintings. Link: https://www.aliyundrive.com/s/xAoLuHTP4Li/folder/62c7f16b82011dc014ff4d3bb3c5e82fb4c309af. Source: Figshare Website. URL: https://doi.org/10.6084/m9.figshare.25511146.v1. Search date: 25 February, 2024. Copyright: Creative Commons Attribution 4.0 International (CC BY 4.0). Description: We found some ancient Chinese and European fine art paintings shared by Kailin Huang under CC BY 4.0 from the Figshare data sharing platform. The author acknowledges that these works are in the public domain and are in accordance with the ’Berne Convention for the Protection of Literary and Artistic Works. ’Chinese Natural History Paintings’, a series of books that are used in Table 4 and Fig 4 of our manuscript. Citations: Huang, Kailin (2016). Window on Culture: Appreciation of Chinese and Western Painting Art. figshare. Dataset. https://doi.org/10.6084/m9.figshare.25511146.v1.

**Fig 6:**
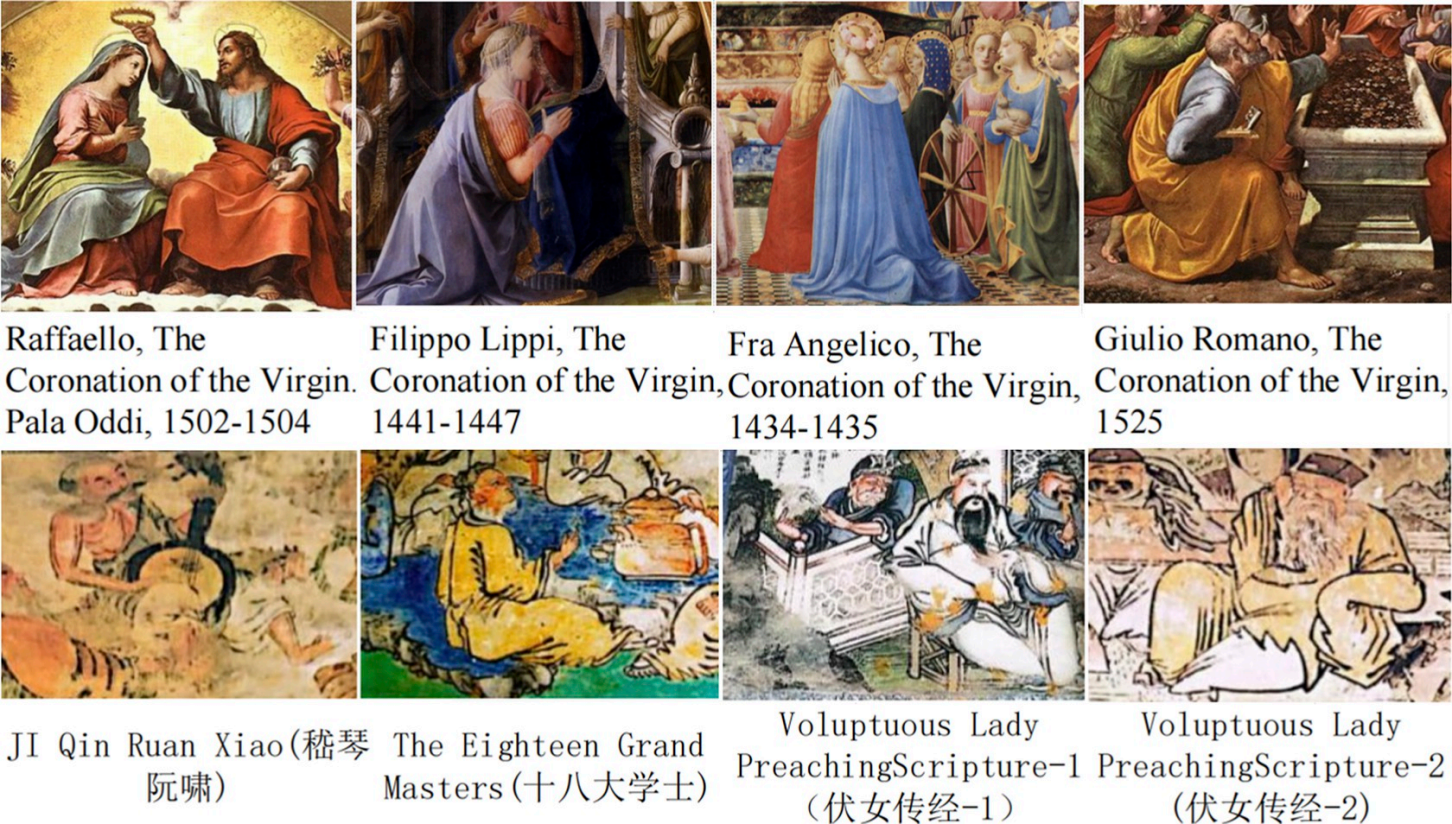
Color contrast between western paintings and Chinese traditional murals. Western paintings: source from https://doi.org/10.3390/rel13121145. Copyright: Creative Commons Attribution 4.0 International (CC BY 4.0). Chinese traditional murals: source from https://doi.org/10.1186/s40494-023-01069-1. Copyright: Creative Commons Attribution 4.0 International (CC BY 4.0). Use situation: use the original picture without any modification.

In **Fig 5A**, the serial numbers A-Q refer to the seventeen albums of Chinese Natural History Paintings, showing the use of colors of costumes of the characters in the books. Although the colors pink, yellow, and indigo (close to pure color) are exceptionally obvious, secondary colors such as purple, light blue, dark blue, and brown occupy more than 50%, which implies that the people of the underclass constituted the main group of characters in Guangzhou export paintings in the Qing Dynasty. It is also consistent with the above statistics on the number of these characters. **Fig 5B** shows the summed-up percentages of the colors in **Fig 5A**, in which the two primary colors, red and indigo, take up an important proportion, reflecting that the use of colors in Qing dynasty export paintings still maintains its tradition. The three secondary colors blue, violet, and brown are preferred by Western art for their high saturation and purity, and take up a certain proportion, indicating the merging of the use of colors in Chinese and Western art. It is demonstrated in the comparison of four Western paintings in **Fig 6**. Being a type of customized artistic commercial that was sold in foreign markets, the unique social attributes of export paintings practice as carriers of the merging of Chinese and Western cultures and arts.

**Fig 5C** analyses the details of the colors used in the costumes of the characters in Guangzhou export paintings, discovering that the use of coordinated and soft secondary colors makes the characters in Guangzhou export paintings of the Qing dynasty distinct differences from the characters in the traditional Chinese paintings of the period that are painted with traditional primary colors (with a pale-looking, solemn and constrained expression) [46] (**Fig 6**). The use of mixed secondary colors on these characters reflects the fact that the painters were not influenced by the feudalism of the time during painting. At the same time, the painters used Western painting skills to represent the light and shade contrast and perspective of the characters’ faces for a more realistic performance, thus integrating the two systems of Chinese and Western painting techniques. It is also an affirmation of the value of human-centered creation and human dignity.

### 3.3 Logic map of the evolution of decorative art of Guangzhou export paintings in the context of trade

As Fallan [47] articulates, from the perspective of cultural history studies, design culture is not an elite culture but a daily one. Contemporary design history transcends the narrative of objects and designers to increasingly embody a history of translation and integration among objects, individuals, and concepts of creation. Export paintings, as artifacts documenting the continuous global cultural interactions of the 18th century, have become integral evidence of the coexistence and integration of Chinese and Western cultural exchanges. Research on the iconography of export paintings enhances understanding of Chinese export art and the history of Sino-foreign cultural exchanges during this period, fostering emotional resonance closely linked to historical contexts. This also opens new research avenues and possibilities for the preservation, inheritance, and sustainable appreciation of Qing Dynasty export painting art.

**Fig 7:**
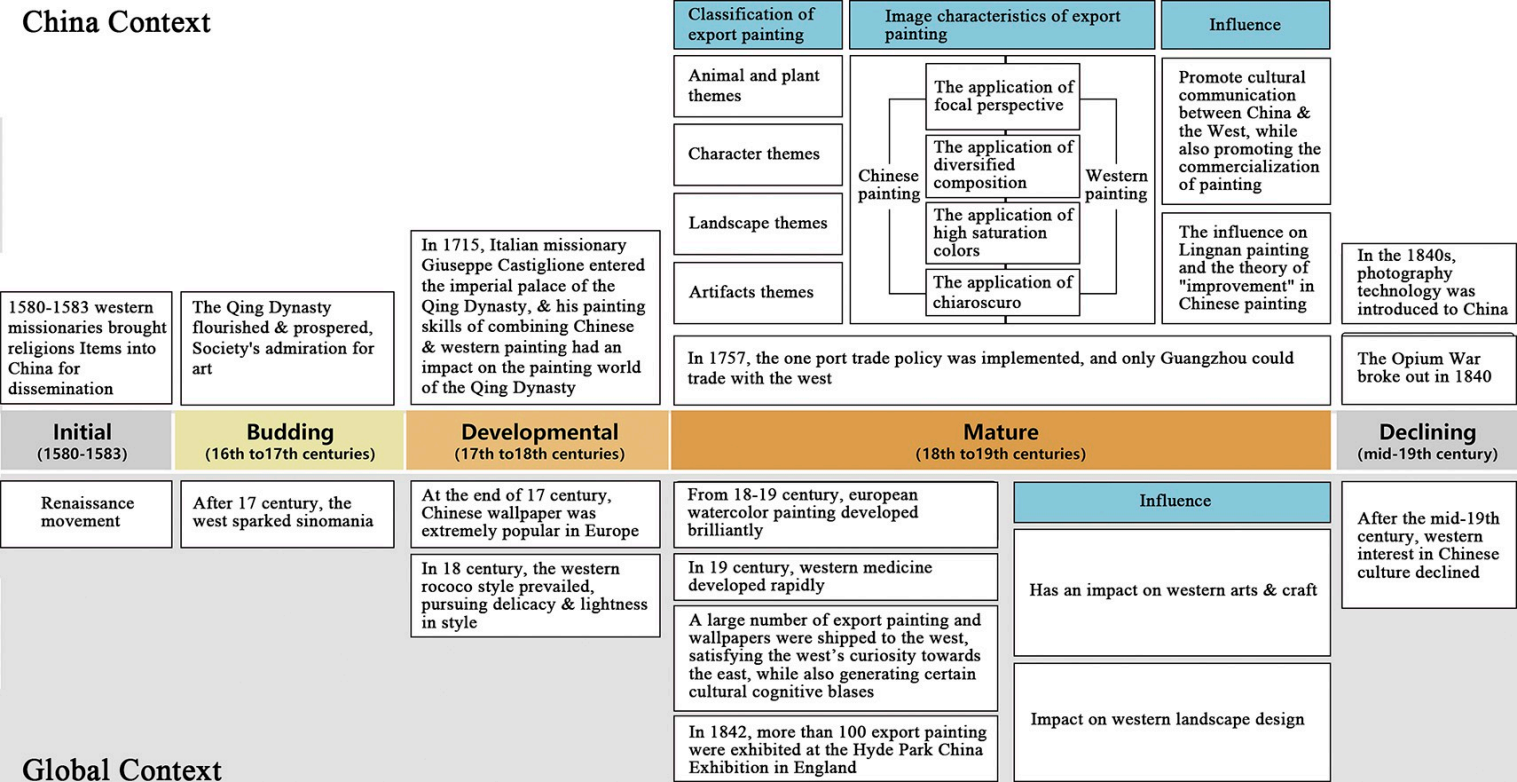
Logic diagram of the evolution of decorative arts in Guangzhou export paintings in the context of trade (Source: Drawn by authors).

**Fig 7** illustrates the developmental logic of Guangzhou export painting art under the trade background, divided into five phases and revealing a dual development trajectory. Initially, Phase One (1580–1583) marks the Renaissance’s preliminary influence on Chinese export painting art, introducing European artistic elements and techniques. Phase Two (16th-17th century) witnessed the "Chinese Art Craze," where the West’s keen interest in Chinese culture encouraged a diversification of artistic styles and themes. In Phase Three (17th-18th century), the arrival of Italian missionaries introduced the Rococo style into the Chinese court, adding delicacy and elegance to artistic expressions. Phase Four (18th-19th century) saw Guangzhou becoming a trade monopoly center, transitioning the role of export paintings in commercial and cultural exchanges from purely artistic to commodified objects. Finally, in Phase Five (mid-19th century), the dual impact of the Opium Wars and photographic technology gradually phased export paintings out of the global market, symbolizing the end of the Western craze for Chinese art.

As these phases progressed, the characteristics of Guangzhou export painting art evolved from abstract → representational → commercialized, exemplified as follows: 1) Composition complexity increased, reflecting the fusion of Chinese and Western art styles; 2) Color application transitioned from traditional to vibrant, showing the adoption and application of Western colors; 3) Subject matter diversified from singular to multifaceted, encompassing a broader range of social life and cultural elements; 4) Color and light/shadow treatment became more refined, indicating technological innovation and the pursuit of visual effects; 5) Artistic methods evolved from imitation to innovation, demonstrating Chinese painters’ absorption and restructuring of Western techniques and artistic concepts; 6) The transition in color usage from primary to mixed intermediate colors; 7) Similarity and homogenization in the elements combined within the paintings. These evolutionary traits not only depict the developmental trajectory of Chinese export art but also its unique position and role in global cultural exchanges.

## 4 Discussions

Export paintings, serving as pivotal mediums of cross-cultural exchange, have provided Western countries with significant insight into the exotic cultures of the East. German artist Beuys [48] posited that such art forms have facilitated the artification and legitimate dissemination of "social dialogue." Set against specific historical backdrops, export paintings stand as tangible evidence of Sino-foreign trade interactions and cross-cultural engagements. As viewers engage with these works, the original referential meanings of garden landscapes and character imagery gradually transform, unveiling the unique construction of Eastern beauty. Notably, in the depiction of character imagery, including the treatment of light and shadow and the precise portrayal of attire, export paintings encapsulate the fusion of Eastern and Western aesthetic principles and painting techniques.

On the aspect of image study of characters, Yu [49] analyses the market’s act on the massive production in Guangzhou of a portrait of the United States’s President Washington and the good-selling in America in the 18th century. Liu [50] takes female characters as an angle of entry to deduce the fact that export paintings had acquired commercial features by studying the relationship between painters and clients. This paper will further investigate the commercialization of the export paintings and extend the discussion to the study of the assembly-line pattern of production, to interpret the essential reason for the duplication of the figure images. Wang [51] holds the opinion that the painting of the characters in export painting combined the techniques of traditional line sketching in Chinese-style painting and light and shade contrast and perspectives in Western-style painting, which is also approved and evidenced by samples 107 and 109 provided by this paper. In this article, we find out that the formation of the Qing Dynasty export paintings was also affected by the local traditional ideologies and the human-centered approach. This painting style, distinct from traditional Chinese painting and purely Western techniques, employs a soft-focus perspective to create a unique dual-language of art. Although this approach deviates from the lofty elegance and creative standards sought in traditional Chinese painting, it caters to Western audiences’ fascination with the mystique of the East through narrative themes, cheerful ambiance, and exotic imagery. The integration of Western composition skills and perspective not only enhances the visual readability of these works but also the authenticity and emotional impact of their narrative content.

On the aspect of studying the use of colors in the characters’ costumes, Huang [52] believes that the designs of Canton costumes were influenced by traditional social institutions. This paper also pays attention to the restrictions on characters’ color of costumes in export paintings cast by the firm social etiquettes. The turning of use from traditional pure colors to mixed colors made prominent rigid hierarchy in Chinese society of the Qing Dynasty (Lin [42] focuses on the aspect of official uniforms in export paintings to interpret the notion of hierarchy), meanwhile, a radiance of humanity is revealed from the export paintings by comparable analyses with the use of colors of western-style paintings of that time.

As ‘commodity art,’ export paintings also embody educational and societal functions. **Table 5** illustrates a consensus among scholars: 1) Export paintings serve as an effective medium for exploring philosophical thoughts, industrial technology, and the history of cultural arts between the East and West, offering researchers a window into understanding cross-cultural exchange and knowledge transfer; 2) They map the relationships within the global trade networks of the 18th and 19th centuries, societal and cultural shifts, and the local geopolitical dynamics, providing a unique perspective for analyzing international relations and cultural interactions at that time. These studies highlight the significant value of export paintings in terms of their educational and social contributions.

**Table 5:** Educationality and sociability function of export painting.

Functions	Findings	References
Educationality	Export paintings not only emerge as products of artistic creation but also serve as platforms for cross-cultural education. With their rich content and forms, they offer invaluable perspectives and materials for understanding the social culture, legal concepts, aesthetic trends, and Sino-Western artistic exchanges of the late Qing Dynasty. The complementarity and differences among these research findings collectively underscore the multidimensional value of export paintings as educational tools, highlighting the significance of in-depth studies of these visual documents in enhancing historical-cultural understanding and facilitating cross-cultural exchanges.	[3, 9, 10, 42, 53–55]
Sociability	Functioning as effective mediums for enhancing social capital and fostering cultural exchanges, export paintings reflect their pivotal role in cross-cultural interactions. Research demonstrates how these artworks are manufactured and remanufactured within the global production and consumption chains, revealing their importance in documenting material and socio-cultural changes. Although theoretically, export paintings contribute to constructing and reinforcing national identity, this effect was not fully realized in practice initially. Instead, they more vividly illustrate how global trade and market expansion impact the production and distribution of art.	[16, 56–59]

Source: own study

Educationally, as carriers of history and culture, export paintings present a wealth of knowledge and understanding of past lifestyles to modern society, also serving as crucial visual aids in art and history education. Socially, these works not only reflect the social customs of their time but also act as bridges fostering understanding and respect between different cultures. By depicting everyday life and significant events, export paintings offer viewers a means to reflect on the evolution of social structures and behaviors.

Through the careful portrayal of characters’ three-dimensionality, realism, and liveliness, export paintings facilitated the trend of Western painting techniques spreading eastward, accelerating the interaction and integration of Eastern and Western cultures. The development of this art form not only enriched the diversity of artistic expression but also built bridges for mutual understanding and exchange between Eastern and Western cultures, showcasing innovation and adaptability in cross-cultural art exchanges. Therefore, export paintings hold an indispensable position in the history of art and cultural exchanges, witnessing cultural collisions and integrations during historical periods while also reflecting the evolution of aesthetic concepts and artistic techniques from a cross-cultural perspective.

The samples that this paper studies come mainly from 6 volumes of the Chinese Natural History Paintings (17 volumes in total), and most of the character samples are concentrated on the Tea Processing and Porcelain Trading volumes, thus possessing limitations on the width and depth of analyses. In addition, compared with Lin [50] and Wang’s [55] studies, this paper also lacks in-depth analyses of the painters’ styles, occupations of the characters, classification of costumes as well as their impacts on the art of export paintings. Finally, this paper still stays in the qualitative description based on the literature research and lacks the assessment of the degree of influence in sorting out the influencing factors of the decorative art of export painting.

## 5 Conclusions

The study indicates that: 1) most of the characters in the Qing Dynasty export paintings are standing and are more likely laboring people demonstrating a negative state of mind. Most officials, foreign merchants, and opera performers express delighted expressions; 2) the scarcity of female characters in export paintings reflects their marginalized status in the social structure while serving as a foil to the dominance of males in the society; 3) export paintings possess commodity features, and the mechanical, assembly-line pattern of production is the key factor that results in the similarities of the characters in the paintings; 4) Qing Dynasty export paintings’ choosing subjects from life with realistic portraying of characters’ expressive manner, with a part of the civilian characters representing inspiring spirits and the use of mixed colors for the costumes are all making this art glamoured with a human-centered touch; 5) Guangzhou export painting decorative art evolution of the development of the dual development of Chinese and Western fusion of veins.

As customized art commodities, export paintings are a micro reflection of Guangzhou’s unique trade culture and social power, through which we can examine the social power structure and ideology of the region by using it as a lens of cultural symbols. The similarities of the characters and the extensive use of mixed colors on their costumes in export paintings disclose the complex intertwining of the development of trading and economics in Guangzhou from the late 18th to the mid-to-late 19th century, as well as the subtle dynamics of power, capital, and art in the process. These precious paintings that absorbed advantages both from Western-style and Chinese-style painting techniques, became a historical record of the coexistence of Chinese and Western cultures and an aesthetics mirror of the Qing Dynasty export arts. Overlooking from a view of global culture and trade networking, the Qing Dynasty Guangzhou export paintings satisfy the customers’ curiosities for foreign cultures and their reverie of “oriental civilization”. Our team will put further effort into researching the correlation between the characterization of the figures and the layout and composition of the scenes of the export paintings.

## Supporting information

S1 Appendix (ZIP)
